# Teaching Medical Students Rapid Ultrasound for shock and hypotension (RUSH): learning outcomes and clinical performance in a proof-of-concept study

**DOI:** 10.1186/s12909-024-05331-3

**Published:** 2024-04-02

**Authors:** Lukas Martin Müller-Wirtz, William M. Patterson, Sascha Ott, Annika Brauchle, Andreas Meiser, Thomas Volk, Ulrich Berwanger, David Conrad

**Affiliations:** 1 Saarland University, Anaesthesiology, 66424 Homburg, Saarland, Germany; 2 Department of Anaesthesiology, Intensive Care and Pain Therapy, Saarland University Medical Center, 66424 Homburg, Saarland, Germany; 3 Outcomes Research Consortium, Department of Anesthesiology, Cleveland Clinic, 44195 Cleveland, OH USA

**Keywords:** Medical education, Ultrasonography, Emergency medicine, Critical care, POCUS, RUSH

## Abstract

**Background:**

Point-of-care ultrasound (POCUS) is a critical diagnostic tool in various medical settings, yet its instruction in medical education is inconsistent. The Rapid Ultrasound for Shock and Hypotension (RUSH) protocol is a comprehensive diagnostic tool, but its complexity poses challenges for teaching and learning. This study evaluates the effectiveness of a single-day training in RUSH for medical students by assessing their performance in clinical scenarios.

**Methods:**

In this prospective single-center observational proof-of-concept study, 16 medical students from Saarland University Medical Center underwent a single-day training in RUSH, followed by evaluations in clinical settings and on a high-fidelity simulator. Performance was assessed using a standardized scoring tool and time to complete the RUSH exam. Knowledge gain was measured with pre- and post-training written exams, and diagnostic performance was evaluated with an objective structured clinical examination (OSCE).

**Results:**

Students demonstrated high performance in RUSH exam views across patients (median performance: 85–87%) and improved scanning times, although not statistically significant. They performed better on simulators than on live patients. Written exam scores significantly improved post-training, suggesting a gain in theoretical knowledge. However, more than a third of students could not complete the RUSH exam within five minutes on live patients.

**Conclusions:**

Single-day RUSH training improved medical students’ theoretical knowledge and simulator performance but translating these skills to clinical settings proved challenging. The findings suggest that while short-term training can be beneficial, it may not suffice for clinical proficiency. This study underscores the need for structured and possibly longitudinal training programs to ensure skill retention and clinical applicability.

**Supplementary Information:**

The online version contains supplementary material available at 10.1186/s12909-024-05331-3.

## Introduction

Since its introduction to the clinical armamentarium in the early 2000s, point-of-care ultrasound (POCUS) has matured into an essential diagnostic tool in perioperative, emergency, and intensive care settings [1–4]. Despite its broad clinical applicability, formal ultrasound instruction in medical education has been scarce and highly variable [5–8]. For example, only half of US medical schools have implemented POCUS training in their curricula [9]. Therefore, structured training in POCUS across medical education is needed.

POCUS is a quick, non-invasive, bedside exam that is useful in the rapid assessment of critically ill patients [10, 11]. Several algorithms were developed to standardize ultrasound examinations and to create a common language to communicate diagnostic signs. One such example is the Focused Assessment with Sonography for Trauma (FAST) exam, used to evaluate pericardial, intraperitoneal, and pleural spaces for free fluid [12].

While FAST is easy to perform, it lacks the ability to diagnose several non-traumatic causes of hemodynamic instability that do not necessarily result in free fluid in the peritoneal cavity. Rapid Ultrasound for Shock and Hypotension (RUSH) is a separate diagnostic algorithm that includes additional sonographic views to assess a greater number of clinically relevant causes for hemodynamic instability than FAST [13, 14]. RUSH has demonstrated good diagnostic accuracy [15], but is associated with lower teaching success than FAST, most likely due to its increased complexity (9–11 views versus 4–5) [16]. Therefore, there is further need to assess the learning outcomes of teaching RUSH in medical students.

Ultrasound teaching courses often use simulation; however, in practice patients are often more difficult to scan with common conditions, such as obesity, impeding scanning performance [17]. Therefore, the evaluation of learning outcomes should assess scanning performance on real patients in a clinical setting.

This prospective observational study assessed the utility of a single-day training in RUSH with a focus on real-world scanning performance. We evaluated performance via both a novel performance score for RUSH and by measuring the time needed to perform the examination. Secondarily, we evaluated students’ gain in knowledge after course participation as well as their diagnostic performance with an objective structured clinical examination (OSCE) using an ultrasound simulator.

## Methods

This study was approved by the responsible ethics committee (approval date: 24th January 2022, reference number: 03/22, Ethikkommission der Ärztekammer des Saarlandes, Saarbrücken, Germany). Written informed consent was obtained from each study subject. Our report adheres to the STROBE criteria and the study protocol is provided in supplementary file 1.

### Study design

**Fig. 1:**
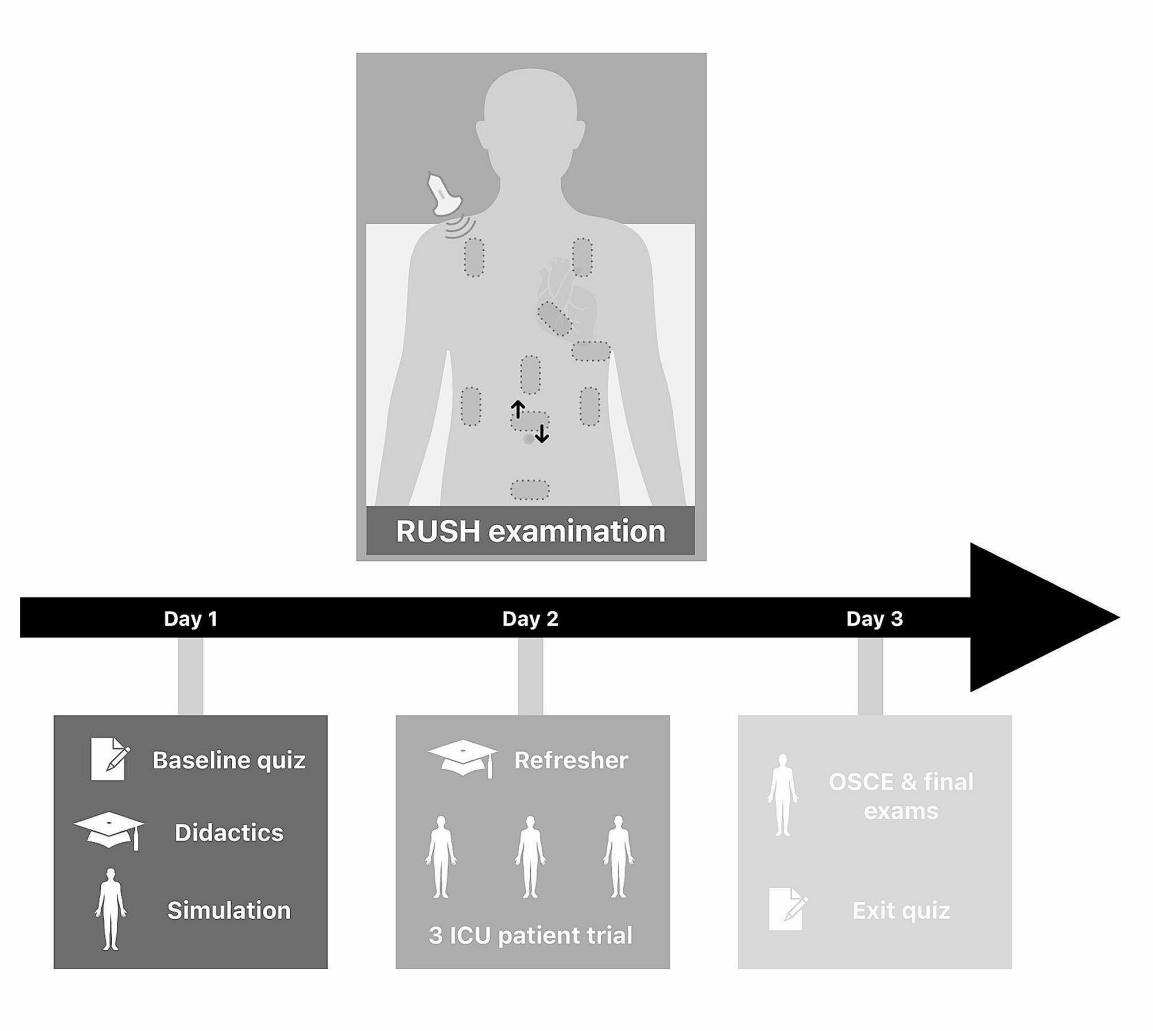
Flow chart of the educational course structure. After one day of instruction, students were assessed in a variety of simulated and clinical environments over the following two course days

This was a prospective single-center observational proof-of-concept study to evaluate the clinical performance of medical students and teaching outcomes after a single-day training in ultrasound for medical emergencies according to the RUSH protocol (Fig. 1). Ultrasound instruction and subsequent evaluation lasted three days and was conducted with 16 medical students at the Saarland University Medical Center, Saarbrücken, Germany. All medical students were eligible to participate in this study, except for students with extensive prior ultrasound experience. The final course structure was optimized based on the feedback obtained during a preceding condensed course performed with 11 final-year medical students of our department. The condensed course included all educational parts within one day but did not include practical and theoretical exams.

### Course structure

The overall course structure is visualized in Fig. 1. Participants received a pre-reading at least one week prior to the course providing a detailed description of the RUSH exam [14]. The team of academic tutors was composed of one anesthesiology resident and three board-certified anesthesiologists, all of whom had extensive training and clinical experience in perioperative and intensive care ultrasound diagnostics. At the beginning of the course, entry knowledge was assessed by a written exam (Supplementary File 2).

The first day of training included 4 h of theoretical lectures and practical demonstrations of the RUSH exam in the morning. This was followed by 4 h of practical scanning exercises. For the practical exercises, participants were divided in 4 groups with 4 students each to perform the RUSH exam for 3 h amongst each other and 1 h on a high-fidelity ultrasound simulator (Simbionix Ultrasound Mentor, Surgical Science, Göteborg, Sweden). Every participant was scanned at least three times by every group member, resulting in at least 9 scans performed by each participant on another participant. The groups rotated every hour to a new tutor with a break of 10 min between rotations. All groups spent 1 h scanning the ultrasound simulation model. At least 2 scans were performed on the simulator; first with normal anatomy and second with a simulated pathology. Every participant had to complete at least one of the preinstalled simulated training scenarios for the detection of typical pathologies with the RUSH exam (e.g., pulmonary effusion, cardiac tamponade, abdominal bleeding). All scans were performed unblinded to the group with each participant contributing to an open problem-based learning discussion when difficulties with displaying the required ultrasound views originated. Tutors provided oral as well as hands-on feedback to guide the participants. All participants were able to perform the RUSH exam on healthy subjects within 5 min at the end of course day 1.

On the second day, a short recapitulation of the RUSH exam (about 10 min) preceded the evaluation of practical scanning performance in clinical scenarios. Participants performed the RUSH exam on patients in the intensive care unit (ICU) or postoperative recovery room. Patients with extensive coverage of the scanning areas with a surgical dressing or those that declined participation were excluded and no patient-specific data were collected. Participants were encouraged to perform 3 RUSH exams each on a different patient within 5 min. A maximum scanning time of 10 min was granted per scan and performance was rated based on standardized criteria (Supplementary File 3 and 4). After completion of the scan or after reaching the maximum scanning time, oral and hands-on feedback was provided to improve those ultrasound views that the participant found challenging. No feedback or advice was provided during the first scanning attempt on each patient.

On the third day, the practical performance of the participants was evaluated by an objective structured clinical examination (OSCE) on an ultrasound simulator (Supplementary File 5). Exit theoretical knowledge was evaluated through a repetition of the written entry exam (Supplementary File 2).

### Outcomes

Participants’ (1) scanning performance score and (2) time needed to complete the examination in a clinical setting were co-primary outcomes in this analysis. A review of the literature during the planning phase of the study identified potential assessment tools for ultrasound skills (e.g., Objective Structured Assessment of Ultrasound Skills (OSAUS) [18], Ultrasound Competency Assessment Tool (UCAT) [19]). However, these tools did not include a detailed rating of scanning performance and time and were thus deemed unsuitable to answer our study aims. We therefore invented a new performance score for the RUSH exam similar to a previous scoring system used to evaluate simulation-based training of thoracentesis in medical students [20]. Although we did not formally validate our score, we performed test runs during the preceding course with final-year medical students to confirm the applicability of our score.

### Primary outcome 1: performance score

Each ultrasound examination was scored based on a standardized protocol by supervising physicians (Supplementary File 4). At the discretion of the evaluating physician, views that were not possible to obtain (e.g. due to dressings) were removed from the maximum number of achievable points. Each possible ultrasound view included in the RUSH protocol was rated as “fully acquired” (2 points), “partially acquired” (1 point), “not acquired” (0 points) or “not possible”. Rating was guided by predefined objective rating criteria for each ultrasound view (Supplementary File 3). Only one examination was performed and scored per patient. Results were expressed as the percentage of achieved points out of all achievable points.

### Primary outcome 2: performance time

Performance time was defined as the time needed to complete all views of the RUSH exam, measured to the second. Timing was stopped as soon as students captured each of the 9 graded views or the student indicated they had completed the exercise to the best of their ability.

### Secondary outcome 1: score in the practical exam (OSCE)

Obtainable scores in the OSCE were composed of scores related to scanning performance (equivalent to primary outcome 1: performance score) and scores related to the diagnostic and documentation skills of the student during simulation. In addition to rating practical scanning performance on the high-fidelity ultrasound simulator, students were prompted to make a diagnosis for simulated case scenarios based on the views obtained during the RUSH exam. Students also wrote a medical report describing their findings. These reports were rated by evaluators as “good” (2 points), “moderate” (1 point) or “insufficient” (0 points). The result was expressed as a percentage of the maximum achievable points (Supplementary File 5).

### Secondary outcome 2: scores in a written exam

A written exam was performed both before and after the course in a large auditorium with each student individually logging into the institutional digital online examination platform (Moodle, Saarland University, Germany); time was restricted to 25 min. The exam consisted of 16 predominantly multiple-choice questions (Supplementary File 2). The results were expressed as a percentage of the maximum achievable points. The correct answers were neither communicated nor discussed individually during the course before the final exam, and the exams were performed 5 days apart.

### Statistical analysis

Data were collected with Excel 2019 (Microsoft, Redmond, USA). Statistical analyses were carried out with R (R Core Team, 2023) using the tidyverse package (R Core Team 2023; Wickham et al. 2019). Data are presented as means (SD), medians (interquartile range), or frequencies (percentages) as appropriate. Results of performance-related scores are expressed as the percentage of achieved points of the achievable total points. We performed non-parametric pairwise comparisons with the Wilcoxon rank-sum or signed rank test, which were adjusted for multiple comparisons. A two-sided *p* < 0.05 was considered statistically significant. Due to the descriptive nature of this study, no a priori sample size estimation was performed. The highest number of participants possible was included in this study based on available teaching resources and considerations on suitable group sizes.

## Results

The study participants’ characteristics are presented in Table 1.

**Table 1:** Study participants’ characteristics

Number of participants	*n* = 16
**Age (years)**	25 [20, 35]
**Sex (m/f)**	7 (44%) / 9 (56%)
**Semester of medical education**	
1	1 (6%)
5	3 (19%)
6	1 (6%)
7	2 (13%)
8	3 (19%)
9	5 (31%)
11	1 (6%)
**Stage of medical education**	
Pre-clinical	1 (6%)
Clinical	14 (88%)
Practical year	1 (6%)
**Previous exposure to ultrasound**	1 (6%)

Data are presented as median (IQR) or frequencies (percentages)

### Primary outcome 1: performance score

**Fig. 2:**
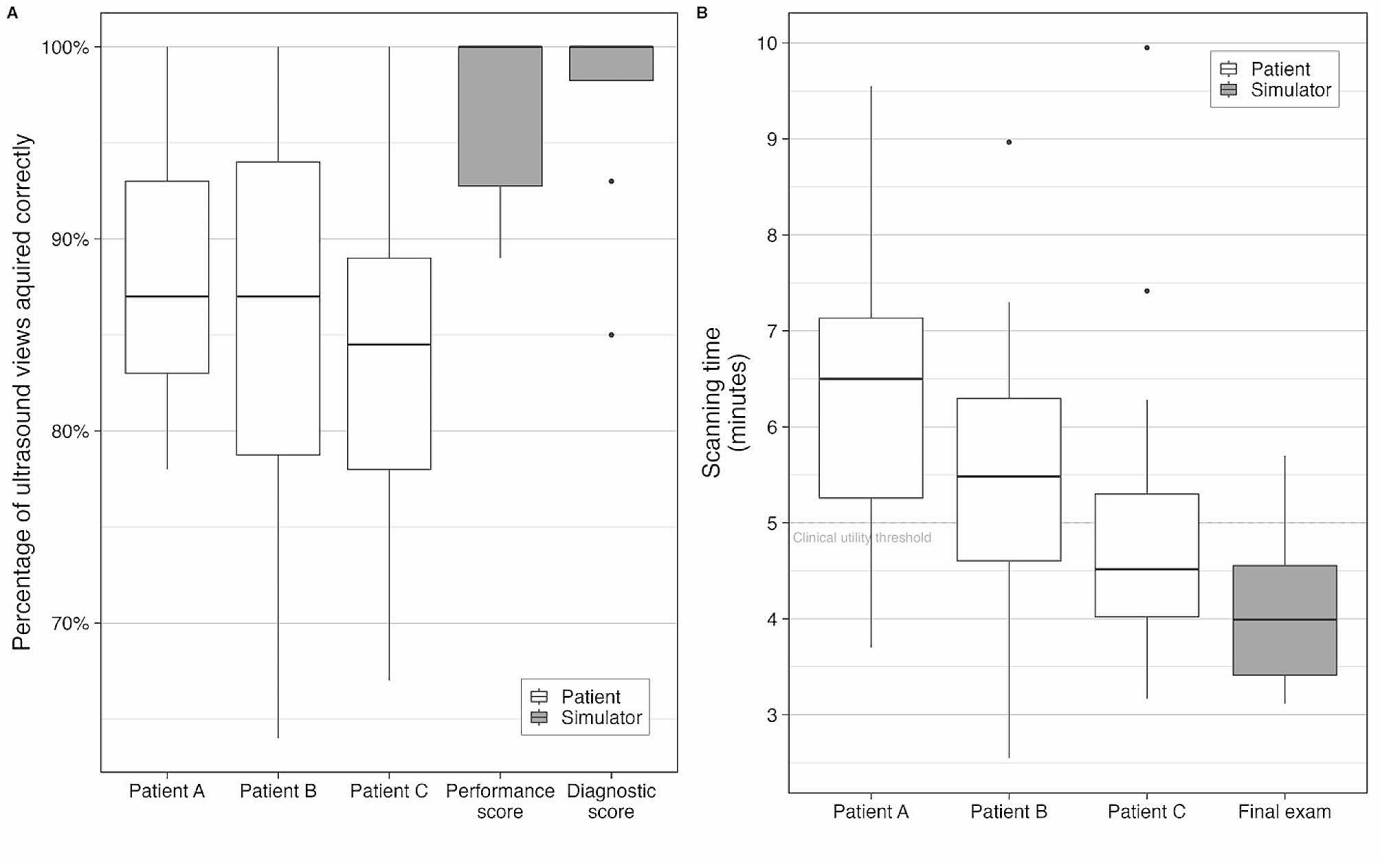
Scanning performance and time. Panel **A**: Participants performed equally well on all three patients and significantly better on simulators than on real patients. Panel **B**: Participants had similar scanning times on all patients but were significantly faster on the simulator. The dashed horizontal line indicates the time limit for clinical utility (as determined by the investigators)

Participants performed equally well on each of the three patients and were able to obtain most of the RUSH exam views on each of the three patients (median [interquartile range (IQR)] performance in patient A: 87 [83, 93] %; patient B: 87 [79, 94] %; and patient C: 85 [78, 89] %, *p* = 0.554; Fig. 2A).

### Primary outcome 2: performance time

Participants were able to complete the RUSH exam in a similar amount of time for each of the three live patients. Students took a median [IQR] of 6 min (m) 30 s (s) [5 m 16 s, 7 m 8 s] on patient A, 5 m 29 s [4 m 36 s, 6 m 18 s] on patient B, and 4 m 31 s [4 m 1 s, 5 m 18 s] on patient C. Between the first and third scan (patient A to C), the median difference in scanning time improved by 1 m 59 s — although this difference was not statistically significant (95% confidence interval (95%CI): 21 s, 2 m 37 s; *p* = 0.07; Fig. 2B).

### Secondary outcome 1: score in the practical exam (OSCE)

Median [IQR] scores in the performance section (i.e., obtaining the images) and the diagnostic section (i.e., documentation and diagnosis) of the final OSCE exam were 100 [93, 100] % and 100 [98, 100] %. Participants performed significantly better on simulators than on live patients (*p* < 0.02 for all patients compared to the simulator; Fig. 2A). Participants were significantly faster on the simulator than on their first and second, but not third live patient (patient A versus simulator, *p* = 0.002; patient B versus simulator, *p* = 0.013; patient C versus simulator, *p* = 0.22; Fig. 2B).

### Secondary outcome 2: scores in a written exam

**Fig. 3:**
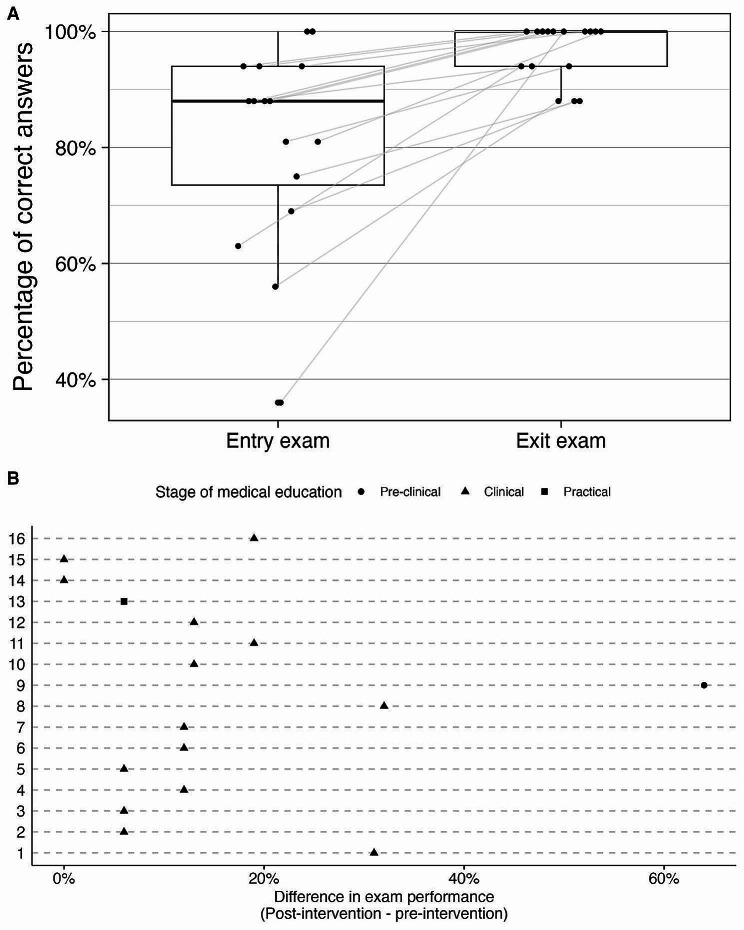
Students’ MC exam performance scores pre- and post-intervention. Panel **A**: Student exam performance improved after instruction (*p* = 0.001, Wilcoxon signed rank test). Panel **B**: Students improved by a median [IQR] difference of 12 [6, 19] %. The outlier (+ 64%) was a first-year student

Participants’ multiple choice written exam scores improved after course participation (*p* = 0.001), with a median [IQR] of 88 [74, 94] % on the entry exam and 100 [94, 100] % on the final exam (Fig. 3A). Performance was widespread on the entry exam ranging from 36 to 100%, but all students scored higher than 85% on the final exam (Fig. 3B). Students had a median [IQR] exam performance improvement of 12 [6, 19]% after course participation.

## Discussion

The RUSH exam can be an invaluable tool in the diagnostic toolbox of physicians in perioperative, emergency, and intensive care settings [13–15, 21]. We evaluated a single-day training in RUSH with a focus on scanning performance under clinical conditions. Despite the brevity of the course, most students were able to capture more than 80% of the ultrasound views of the RUSH exam on each of the three patients with sufficient quality. In addition, two thirds of the students succeeded in performing the RUSH exam within a clinically relevant time frame of 5 min.

While scanning performance did not improve with each patient scan, the required time to complete the RUSH exam decreased, although this improvement was not statistically significant. The study team agreed on a clinical utility threshold of 5-minutes for the RUSH exam. Though most students were able to achieve this by their third patient, more than a third did not complete the exam within that time. Our course participants performed at least 14 RUSH scans prior to the final exam; 9 on healthy subjects, 2 on a simulator, and 3 on patients. To reach reasonable scanning and diagnostic skills, a much higher number of repetitions (50–75) may be needed to master ultrasound examinations used in emergency medicine [22]. However, with reduced complexity of ultrasound examinations, learning plateaus have been seen as early as after 10 to 15 repetitions [23]. Although the RUSH exam requires views from several different body areas (i.e., cardiac, abdominal, and lung ultrasound), it only includes a subset of the most important ultrasound views for each body region, which suggests that a lower number of repetitions is needed to reach a learning plateau– at least in comparison to more detailed examinations. Though our course has been a sufficient primer for the RUSH exam in medical students, many more scans are likely required to master the RUSH exam.

Medical students were slower and performed worse on real patients than on the simulator. While all but one student completed the exam in under 5 min on the simulator, a third needed longer on real patients. This reveals current limitations of ultrasound simulators in refactoring real-world clinical conditions. However, while ultrasound simulators do not replace the real-world experience, they are useful tools to demonstrate principles of ultrasound examinations and gain early learners’ ultrasound skills before approaching real patients [24–30]. Training to mastery on the simulator was found to lower the repetitions needed to master abdominal ultrasound examinations on real patients in 25 first-year residents randomized to simulation-based or conventional clinical training [25]. Simulation-based training thus helps to shorten the steep initial part of a trainees’ learning curve, while lowering the burden on patients resulting from initial scanning attempts by ultrasound novices.

The ability of simulator models to discriminate in proficiency of scanning skills may vary substantially. As such, a recent report showed that a simulation-based assessment of scanning skills discriminated reasonably well between the performance of novices and experts in abdominal ultrasound [31]. In contrast, most participants of our course scored 100% on the simulator. The high median performance of students on our simulator suggests that the model is too easy to scan and fails to provide enough of a distribution to allow for discrimination in scanning skills. Given the significant deviation between performance under clinical and simulated conditions, our results suggest that ultrasound scanning skills should be evaluated on live patients or at least human models to obtain meaningful estimates of scanning performance.

Participation in our course led to a significant gain in theoretical knowledge assessed by a written exam before and after the course– an important tool for quality assurance of the educational value of our course. The fact that some but not all students scored 100% in both entry and final exams suggests reasonable discrimination amongst the performance of the students. Scores on the written exam increased by a relative median difference of 14%, which is lower when compared to a similar report on a course in RUSH and eFAST [16]. In the study of Cevik et al., scores in written exams more than doubled after a course in RUSH in medical final-year students (RUSH: 166%, eFAST: 114% of relative increase in performance) [16]. Also in contrast to our findings, only 47% of students passed the exam for RUSH compared to 79% passing the exam for eFAST [16]. The better performance in the written exams in our study could be explained by the fact that we provided a pre-reading script, leading to a higher baseline knowledge. In addition, our course included 4 h (versus 1) of theoretical lectures, 4 h (versus 2) of practical scanning training, simulator training, and clinical hands-on scanning sessions [16]. The increase in scores after participation in our course suggests that the amount of educational content and hands-on experience was sufficient to achieve a reasonable gain in theoretical knowledge.

The core educational components of our course, such as lectures and hands-on scanning time on participants and the simulator, were conducted in a single day. Reasonable improvement in learning outcomes and clinical performance suggests that the course length was sufficient as a primer for most students to master the RUSH exam. Similarly, Cevik et al. reported a high 84% success rate in passing an OSCE for eFAST after a 1-hour theoretical lecture and 2-hours of practical scanning in 54 final-year medical students [32]. In contrast, Boniface et al. demonstrated that a longitudinal curriculum for internal medicine residents resulted in considerably better skill retention for ultrasound procedures compared to a single-day workshop, suggesting that ongoing education may be more effective for long-term competency [33]. Future studies could explore longitudinal follow-up to evaluate long-term skill retention, or the inclusion of a group exposed to longitudinal refresher courses. Taken together, short-term learning outcomes appear to be reasonably good after a single-day structured training in the RUSH exam, but long-term skill retention remains unclear and could benefit from longitudinal inclusion of refresher courses in academic curricula.

### Limitations

This study evaluated students’ performance under clinical conditions, which directly mirrors the real-world scenarios where the RUSH exam would be employed; however, this study does have limitations which merit attention. First, all medical students were from a single institution, possibly limiting the external validity of the results; however, we did enroll students from different semesters of clinical training. Second, the use of a single-day training session as the intervention may not be sufficient to produce lasting competency in the RUSH exam. Retention of skills over time could be a factor more crucial for clinical applicability. A blend of longitudinal training with periodic reinforcement may emerge as a superior strategy. Third, the small study population and the fact that students had to actively apply for the course, making them a highly motivated sub-population of medical students, severely limits generalizability of our results. Finally, the scoring metric we used to evaluate performance were not previously validated.

## Conclusion

Medical students showed satisfactory performance in both theoretical and practical evaluations following a one-day RUSH training session. However, a notable disparity emerged between simulated and actual scanning environments, with worse performance on real patients. This study enhances our understanding of the effectiveness of brief RUSH training in medical students, especially its applicability in real clinical scenarios. However, critical inquiries persist regarding long-term skills retention and the adaptability of the training program across diverse educational and clinical settings. Future investigations should prioritize addressing these gaps, potentially through multi-institutional or longitudinal studies, to offer a more comprehensive assessment of RUSH exam training’s impact in medical education.

### Electronic supplementary material

Below is the link to the electronic supplementary material.

Supplementary Material 1

Supplementary Material 2

Supplementary Material 3

Supplementary Material 4

Supplementary Material 5

## Data Availability

Data and the instructional slide deck (5 presentations: introduction, basics, shock, RUSH, ultrasound pathology quiz) are available from the corresponding author upon reasonable request. The R code of the statistical analysis can be found in the GitHub online repository of WMP: https://github.com/pattwm16/rushpro-us.

## References

[1] Díaz-Gómez JL, Mayo PH, Koenig SJ (2021). Point-of-Care Ultrasonography. N Engl J Med.

[2] Whitson MR, Mayo PH (2016). Ultrasonography in the emergency department. Crit Care.

[3] Ramsingh D, Bronshteyn YS, Haskins S, Zimmerman J. Perioperative point-of-care ultrasound: from concept to application. Anesthesiology. 2020;:908–16. 10.1097/ALN.0000000000003113 31977521

[4] Campbell SJ, Bechara R, Islam S (2018). Point-of-care Ultrasound in the Intensive Care Unit. Clin Chest Med.

[5] Wolf R, Geuthel N, Gnatzy F, Rotzoll D (2019). Undergraduate ultrasound education at german-speaking medical faculties: a survey. GMS J Med Educ.

[6] Bahner DP, Goldman E, Way D, Royall NA, Liu YT (2014). The state of ultrasound education in U.S. Medical schools: results of a national survey. Acad Med.

[7] Feilchenfeld Z, Dornan T, Whitehead C, Kuper A (2017). Ultrasound in undergraduate medical education: a systematic and critical review. Med Educ.

[8] Rajamani A, Shetty K, Parmar J, Huang S, Ng J, Gunawan S (2020). Longitudinal competence programs for Basic Point-of-care ultrasound in critical care: a systematic review. Chest.

[9] Russell FM, Zakeri B, Herbert A, Ferre RM, Leiser A, Wallach PM (2022). The state of point-of-care Ultrasound Training in Undergraduate Medical Education: findings from a National Survey. Acad Med.

[10] Shokoohi H, Boniface KS, Pourmand A, Liu YT, Davison DL, Hawkins KD (2015). Bedside ultrasound reduces diagnostic uncertainty and guides resuscitation in patients with undifferentiated hypotension. Crit Care Med.

[11] Pontet J, Yic C, Díaz-Gómez JL, Rodriguez P, Sviridenko I, Méndez D et al. Impact of an ultrasound-driven diagnostic protocol at early intensive-care stay: a randomized-controlled trial. Ultrasound J. 2019;11. 10.1186/s13089-019-0139-2 PMC6783485 31595353

[12] Pace J, Arntfield R (2018). Focused assessment with sonography in trauma: a review of concepts and considerations for anesthesiology. Can J Anesth.

[13] Seif D, Perera P, Mailhot T, Riley D, Mandavia D. Bedside ultrasound in resuscitation and the rapid ultrasound in shock protocol. Crit Care Res Pract. 2012;2012. 10.1155/2012/503254 PMC3485910 23133747

[14] Weingart SD, Duque D, Nelson B. The RUSH exam: Rapid Ultrasound for Shock and Hypotension. EMCrit Project. 2008. https://emcrit.org/rush-exam.

[15] Bagheri-Hariri S, Yekesadat M, Farahmand S, Arbab M, Sedaghat M, Shahlafar N (2015). The impact of using RUSH protocol for diagnosing the type of unknown shock in the emergency department. Emerg Radiol.

[16] Cevik AA, Cakal ED, Abu-Zidan F. Point-of-care Ultrasound Training during an Emergency Medicine Clerkship: a prospective study. Cureus. 2019;11. 10.7759/cureus.6118 PMC6844539 31723483

[17] Brahee DD, Ogedegbe C, Hassler C, Nyirenda T, Hazelwood V, Morchel H (2013). Body mass index and abdominal ultrasound image quality: a pilot survey of sonographers. J Diagn Med Sonography.

[18] Todsen T, Tolsgaard MG, Olsen BH, Henriksen BM, Hillingsø JG, Konge L (2015). Reliable and valid assessment of point-of-care ultrasonography. Ann Surg.

[19] Bell C, Hall AK, Wagner N, Rang L, Newbigging J, McKaigney C (2021). The Ultrasound Competency Assessment Tool (UCAT): development and evaluation of a Novel competency-based Assessment Tool for Point-of-care Ultrasound. AEM Educ Train.

[20] Jiang G, Chen H, Wang S, Zhou Q, Li X, Chen K et al. Learning curves and long-term outcome of simulation-based thoracentesis training for medical students. BMC Med Educ. 2011;11. 10.1186/1472-6920-11-39 PMC3144014 21696584

[21] Perera P, Mailhot T, Riley D, Mandavia D (2010). The RUSH exam: Rapid Ultrasound in SHock in the evaluation of the critically lll. Emerg Med Clin North Am.

[22] Blehar DJ, Barton B, Gaspari RJ (2015). Learning curves in emergency ultrasound education. Acad Emerg Med.

[23] Breunig M, Hanson A, Huckabee M (2023). Learning curves for point-of-care ultrasound image acquisition for novice learners in a longitudinal curriculum. Ultrasound J.

[24] Lewiss RE, Hoffmann B, Beaulieu Y, Phelan MB (2014). Point-of-care Ultrasound Education. J Ultrasound Med.

[25] Østergaard ML, Rue Nielsen K, Albrecht-Beste E, Kjær Ersbøll A, Konge L, Bachmann Nielsen M (2019). Simulator training improves ultrasound scanning performance on patients: a randomized controlled trial. Eur Radiol.

[26] Østergaard ML, Ewertsen C, Konge L, Albrecht-Beste E, Bachmann Nielsen M (2016). Simulation-based abdominal ultrasound training– a systematic review. Ultraschall Der Medizin-European J Ultrasound.

[27] Stefanidis D, Scerbo MW, Montero PN, Acker CE, Smith WD (2012). Simulator training to automaticity leads to improved skill transfer compared with traditional proficiency-based training: a randomized controlled trial. Ann Surg.

[28] Jensen JK, Dyre L, Jørgensen ME, Andreasen LA, Tolsgaard MG (2017). Collecting Validity evidence for Simulation-Based Assessment of Point‐of‐Care Ultrasound skills. J Ultrasound Med.

[29] Taksøe-Vester LDJS, C (2020). Simulation-based ultrasound training in obstetrics and gynecology: a systematic review and meta-analysis. J Ultrasound.

[30] Simon R, Petrisor C, Bodolea C, Golea A, Gomes SH, Antal O (2024). Efficiency of Simulation-based learning using an ABC POCUS Protocol on a high-Fidelity Simulator. Diagnostics.

[31] Teslak KE, Post JH, Tolsgaard MG, Rasmussen S, Purup MM, Friis ML (2024). Simulation-based assessment of upper abdominal ultrasound skills. BMC Med Educ.

[32] Cevik AA, Noureldin A, El Zubeir M, Abu-Zidan FM (2018). Assessment of EFAST training for final year medical students in emergency medicine clerkship. Turk J Emerg Med.

[33] Boniface MP, Helgeson SA, Cowdell JC, Simon LV, Hiroto BT, Werlang ME (2019). A longitudinal curriculum in Point-Of-Care Ultrasonography improves medical knowledge and psychomotor skills among Internal Medicine residents. Adv Med Educ Pract.

