# Supplementary material for: Teaching Medical Students Rapid Ultrasound for shock and hypotension (RUSH): learning outcomes and clinical performance in a proof-of-concept study
Source: BMC Med Educ. 2024 Apr 2;24:360. doi: 10.1186/s12909-024-05331-3 (PMC10988853; doi:10.1186/s12909-024-05331-3)
Supplement: Supplementary file 1 — Supplementary Material 1 [file 12909_2024_5331_MOESM1_ESM.pdf]

Teaching Medical Students Rapid Ultrasound for Shock and Hypotension (RUSH): Learning Outcomes and Clinical Performance in a Proof-of-concept study

Lukas M. Müller-Wirtz, MD

Andreas Meiser, MD

Thomas Volk, MD

Ulrich Berwanger, MD

David Conrad, MD

Educational Research Project

Department of Anaesthesiology, Intensive Care und Pain Therapy

Saarland University Medical Center and Faculty of Medicine

Table of Contents

***1. Background and Rationale*......3**

***2. Study Objectives*......4**

***3. Methods and Study Design*......5**

A) Study Overview......5

B) Study Population......7

C) Exposures......7

D) Outcomes...... Error! Bookmark not defined.

E) Data Analysis......9

F) Sample Size Considerations......9

***3. References*......10**

1. Background and Rationale

Point-of-care ultrasound (POCUS) has matured to an essential diagnostic tool [1], particularly in perioperative, emergency and intensive care settings [2–4]. However, teaching of ultrasonography in medical school still remains limited and teaching approaches are highly variable [5,6]. Integration of structured trainings for ultrasonography in medical curricula is thus highly needed.

POCUS is quick, non-invasive, and performed at the bedside which is particularly helpful for the assessment of critically ill patients [7,8]. Several algorithms were developed to standardize ultrasound examinations and to create a common language to communicate diagnostic signs. The probably best-established algorithm is Focused Assessment with Sonography for Trauma (FAST) [9]. However, while FAST is easy to perform, it lacks the ability to diagnose several non-traumatic causes for hemodynamic instability. Rapid Ultrasound for Shock and Hypotension (RUSH) complements the FAST algorithm by several sonographic views to assess almost all clinically relevant causes for hemodynamic instability [10,11], shown to provide a good diagnostic accuracy [12]. However, RUSH is associated with lower teaching success than FAST, most probably due to increased complexity [13]. There is thus further need to assess the learning outcomes of teaching RUSH in medical students.

Ultrasound teaching courses often use young and healthy models for practical trainings. However, real patients are often considerably harder to scan; for example, obesity may impede scanning performance [14]. The evaluation of learning outcomes should thus include the assessment of scanning performance under clinical conditions.

This prospective observational study therefore aims to evaluate the learning outcomes of medical students after participating a single-day training in RUSH with a focus on performance under clinical conditions. We will primarily evaluate the clinical performance by a newly created performance score for RUSH and the time needed to perform the examination. Secondary outcomes will be the participants' knowledge before and after the course, performance in a final objective structured clinical examination (OSCE), and the results of a subjective evaluation by the participants.

2. Study Objectives

We propose a prospective observational single-cohort study assessing the learning outcomes of medical student that participate a course for point of care ultrasound for medical emergencies according to the RUSH protocol (Rapid Ultrasound for Shock and Hypotension). The proposed study will have the following aims:

Primary Aim 1

To evaluate clinical scanning performance by a newly created performance score for RUSH over several examination attempts on intensive care or postoperative patients after participating a single-day introductory training course.

Hypothesis. None (descriptive)

Primary Aim 2

To evaluate the time needed to perform RUSH over several examination attempts on intensive care or postoperative patients after participating a single-day introductory training course.

Hypothesis. None (descriptive)

Secondary Aim 1

To evaluate the practical learning success of the participants by an objective structured clinical examination (OSCE).

Hypothesis. None (descriptive)

Secondary Aim 2

To evaluate the theoretical learning success by comparing the theoretical knowledge of the participants before and after course completion.

Hypothesis. We hypothesize that course participation will increase the score achieved in a written exam performed before and after the course.

Secondary Aim 3

To obtain the subjective evaluation by the participants.

Hypothesis. None (descriptive)

3. Methods and Study Design

A) Study Overview

We propose a prospective single-center observational study to evaluate the clinical performance of medical students and teaching outcomes after a single-day training in ultrasound for medical emergencies according to the RUSH protocol (Rapid Ultrasound for Shock and Hypotension). The participants will receive a prereading for the course providing a detailed description of the RUSH protocol. At the beginning of the course, the participants' knowledge will be assessed by a written entry exam. The first day of training will include theoretical lectures, practical demonstrations, and exercises on the simulator and among the participants. On the second day, we will evaluate the practical performance of the participants in carrying out RUSH on patients in the intensive care unit or in the recovery room. On the third day, the practical performance of the participants will be evaluated by an objective structured clinical examination (OSCE) on an ultrasound simulator and the written entry exam will be repeated to evaluate the participants' gain in theoretical knowledge. Additionally, each participant will have to provide a subjective evaluation of the course.

B) Course Overview

Prereadings (approx. 2 – 4 hours):

1) Bowman, Boitnott and Miesemer (2017). THE Point of Care ULTRASOUND HANDBOOK - The RUSH Exam: FOAMed edition. EMSPOCUS. (https://emcrit.org/wp-content/uploads/2018/03/RUSH-FOAMed-Version-w-Corrections-better.pdf)
2) The Pocus Atlas: thepocusatlas.com/shock

Day 1 – Training (6 hours):

- Entry test (20 MC questions)
- Theory: Ultrasound basics; Shock; RUSH protocol; Pathology quiz
- Practical exercises with demonstrations of common pathologies on the simulator
- Practical exercises among the participants

Day 2 – Practice under clinical conditions (about 4 hours per group):

- Short repetition of the theory
- RUSH examinations of intensive care or postoperative patients
- 3 examinations each on a separate patients will be performed

Day 3 – Exams:

- Final practical exam (OSCE)
    - Demonstration of the RUSH protocol on a participant with normal findings
    - Identification of a pathology on the simulator with RUSH
- Repetition of the entry test (20 MC questions)

C) Study Population

Ethical approval will be obtained prior to study execution. Informed consent and data use agreements will be obtained from each participant of the study.

Inclusion criteria

- Medical students willing to participate in the course.
- Medical students within the clinical period of study (lower years preferred).

Exclusion criteria

- Extensive ultrasound experience (e.g., previous participation in equivalent ultrasound courses).
- Final (practical) year medical student.

D) Exposure

The above-described day 1 of an introductory academic teaching course for RUSH.

E) Outcomes

Primary Outcome 1: *Performance score.*

Each ultrasound examination performed on patients will be scored based on a standardized protocol (performance score). The results will be expressed as the percentage of achieved points of the totally achievable points.

Scoring of the ultrasound examination performance

Each ultrasound view included in the RUSH protocol will be rated as "fully acquired" (2 points), "partially acquired" (1 point), "not acquired" (0 points) or "not possible". Views that cannot be scanned, e.g. due to dressings, reduce the maximum number of achievable points. Only one examination will be carried out and scored per patient. For more details, please review the enclosed documents "Performance Score" and "Ultrasound View Rating Criteria".

Primary Outcome 2: *Performance Time.*

The time needed to perform each examination measured accurate to the second.

Secondary Outcome 1: *Score in the practical exam (OSCE)*

The performance score (secondary outcome 1) will be extended by an evaluation of the diagnostic and documentation skills of the participants during simulation.

Additional to the performance score, we will score whether the participant makes the right diagnosis after performing RUSH on a simulator (scoring according to the simulator's program). Furthermore, the participants must write a medical report. The reports are rated as "good" (2 points), "moderate" (1 point) or "insufficient" (0 points). The overall result will be expressed as percentage of the maximum achievable points. For more details, please review the enclosed document "OSCE Checklist".

Secondary Outcome 2: *Score in the written exams*

A written exam will be performed before and after the course. The exam will consist of 20 questions (predominantly multiple choice). The results will be expressed as percentage of the maximum achievable points.

Secondary Outcome 3: *Subjective evaluation by the participants*

Each participant will get an evaluation sheet with several items to score. The overall rating of the course with a scale of 0 to 10 (0 worse and 10 best scoring) will represent the most important tertiary outcome 3. For further detail, please review the enclosed document "RUSHPRO Evaluation Sheet".

F) Data Analysis

Data will be presented as means (SD) or medians (IQR) according to data distribution. $P<0.05$ will be considered statistically significant.

Primary Outcomes 1 and 2:

Performance Score, Performance Time.

The obtained Performance Scores and Performance Times over the attempts will be summarized with a mean for each participant and graphically displayed.

Secondary Outcomes 1 and 3:

Score in the practical exam (OSCE), Subjective evaluation by the participants

The obtained scores and the results of the subjective evaluation will be described in a table or graphical format.

Secondary Outcome 2: *Score in the written exams*

The obtained scores before and after course participation will be compared by an unadjusted bivariate statistical test for paired samples according to the obtained distribution (paired t-test or Wilcoxon signed-rank test).

G) Sample Size Considerations

This is a descriptive study. Thus, no a priori sample size estimation was performed. The highest number of participants possible, regarding available teaching resources, will be included in this study.

4. References

1. Díaz-Gómez JL, Mayo PH, Koenig SJ. Point-of-Care Ultrasonography. Ingelfinger JR, editor. N Engl J Med. 2021;385:1593–602.

2. Whitson MR, Mayo PH. Ultrasonography in the emergency department. Crit Care. Critical Care; 2016;20:1–8.

3. Ramsingh D, Bronshteyn YS, Haskins S, Zimmerman J. Perioperative point-of-care ultrasound: From concept to application. Anesthesiology. 2020;908–16.

4. Campbell SJ, Bechara R, Islam S. Point-of-Care Ultrasound in the Intensive Care Unit. Clin Chest Med. 2018;39:79–97.

5. Wolf R, Geuthel N, Gnatzy F, Rotzoll D. Undergraduate ultrasound education at German-speaking medical faculties: A survey. GMS J Med Educ. 2019;36:1–23.

6. Bahner DP, Goldman E, Way D, Royall NA, Liu YT. The state of ultrasound education in U.S. Medical schools: Results of a national survey. Acad Med. 2014;89:1681–6.

7. Shokoohi H, Boniface KS, Pourmand A, Liu YT, Davison DL, Hawkins KD, et al. Bedside ultrasound reduces diagnostic uncertainty and guides resuscitation in patients with undifferentiated hypotension. Crit Care Med. 2015;43:2562–9.

8. Pontet J, Yic C, Díaz-Gómez JL, Rodriguez P, Sviridenko I, Méndez D, et al. Impact of an ultrasound-driven diagnostic protocol at early intensive-care stay: a randomized-controlled trial. Ultrasound J. Springer Milan; 2019;11.

9. Pace J, Arntfield R. Focused assessment with sonography in trauma: a review of concepts and considerations for anesthesiology. Can J Anesth. Springer US; 2018;65:360–70.

10. Seif D, Perera P, Mailhot T, Riley D, Mandavia D. Bedside ultrasound in resuscitation and the rapid ultrasound in shock protocol. Crit Care Res Pract. 2012;2012.

11. Weingart SD, Duque D, Nelson B. The RUSH Exam: Rapid Ultrasound for Shock and Hypotension [Internet]. EMCrit Proj. 2008. Available from: https://emcrit.org/rush-exam

12. Bagheri-Hariri S, Yekesadat M, Farahmand S, Arbab M, Sedaghat M, Shahlafar N, et al. The impact of using RUSH protocol for diagnosing the type of unknown shock in the emergency department. Emerg Radiol. 2015;22:517–20.

13. Cevik AA, Cakal ED, Abu-Zidan F. Point-of-care Ultrasound Training During an Emergency Medicine Clerkship: A Prospective Study. Cureus. 2019;11.

14. Brahee DD, Ogedegbe C, Hassler C, Nyirenda T, Hazelwood V, Morchel H, et al. Body mass index and abdominal ultrasound image quality: A pilot survey of sonographers. J Diagnostic Med Sonogr. 2013;29:66–72.

5. Attachments

- RUSHPRO Performance Score
- RUSHPRO Ultrasound View Rating Criteria
- RUSHPRO OSCE Checklist
- RUSHPRO Evaluation Sheet (pending)
