# Supplementary material for: Teaching Medical Students Rapid Ultrasound for shock and hypotension (RUSH): learning outcomes and clinical performance in a proof-of-concept study
Source: BMC Med Educ. 2024 Apr 2;24:360. doi: 10.1186/s12909-024-05331-3 (PMC10988853; doi:10.1186/s12909-024-05331-3)
Supplement: Supplementary file 2 — Supplementary Material 2 [file 12909_2024_5331_MOESM2_ESM.pdf]

Rapid Ultrasound for Shock and Hypotension (RUSH)

Drag the labels to the correct location in the image legend.

Aorta

What would be a normal diameter of an aorta?

- 5 cm
- 2.5 cm
- 6 cm
- 3.5 cm
- 8 cm

Bat-Sign

What is the Bat-Sign?

- A sonographic sign of a pneumothorax.
- The visibility of the bat sign excludes a pneumothorax.
- Has the same meaning as the Seashore Sign.
- Is a prognostically unfavorable sign in the context of cardinal decompensation.
- This is the sonographic image that is obtained between two ribs during lung sonography.

B-Lines

What do B-Lines mean?

- Pneumothorax
- Wet lungs/pulmonary edema
- Atelectasis
- Normal artifact with no diagnostic relevance
- Hemothorax

IVC

What is a collapsed inferior vena cava a possible sign of?

- Hypovolemia
- Global heart failure
- Pulmonary embolism
- Cardiac tamponade
- Liver cirrhosis

Respiratory variation in IVC diameter

Respiratory responsiveness of the inferior vena cava means...

- ... circulatory instability.
- ... an insufficient preload on the heart.
- ... should always be interpreted in the patient's overall clinical picture.
- ... too much afterload on the heart.
- ... that the patient has mitral valve regurgitation.

IVC collapse

To examine the respiratory responsiveness of the inferior vena cava (IVC), we have the patient ... three times?

- cough
- inspire
- txhale
- talk
- bear down (as if you were going to defecate)

Pulmonary embolism

What is an echocardiographic sign of possible pulmonary embolism?

- Severe mitral valve regurgitation.
- An enlarged right ventricle compared to the left ventricle (D sign).
- An enlarged left atrium.
- Akinesia of the apex of the left ventricle.
- Aortic valve regurgitation.

Perikarderguss

Which cardiac view is best to rule out pericardial effusion?

- Apical 4 chamber view
- Apical 2 chamber view
- Parasternal long axis
- Parasternal short axis
- Subxyphoid 4 chamber view

Pneumothorax

What is a sonographic sign of a pneumothorax?

- Lung point sign
- Lung pulse sign
- Lung sliding
- B-lines
- C-lines

Pump

Which heart valve can I use to estimate the left ventricular pumping function in the parasternal long axis?

- Aortic valve
- Mitral valve
- Tricuspid valve
- Pulmonary valve
- No heart valves are suitable for this.

Pericardial effusion

When should a cardiac tamponade be punctured as an emergency?

- From 2 cm wide.
- If it was present for a long time and is large.
- If there is diastolic collapse of the right ventricle.
- When it extends circularly around the entire heart.
- If fluid is detectable between liver and right ventricle.

Right Upper Quadrant

Free abdominal fluid in the right upper quadrant usually appears...

- ... at the tip of the liver.
- ... around the gallbladder.
- ... under the kidney.
- ... between liver and kidney.
- ... cranial to the liver.

Shock

What best describes the pathophysiology underlying circulatory shock?

- A severe psychologically stressful event.
- An undersupply of oxygen to the body tissues due to a disruption in the circulation.
- Hypotension that is refractory to treatment.
- The patient is dependent on catecholamines.
- Failure of the heart to pump.

Spine-Sign

What does the spine-sign indicate?

- An extensive pleural effusion.
- Osteoporosis.
- Free abdominal fluid.
- Heart failure.
- A full bladder.

HIMAP

What does HIMAP stand for? Please enter the meaning of each letter in the correct order, separated by a comma and without spaces.

- Type in your answer: …
