# Supplementary material for: Teaching Medical Students Rapid Ultrasound for shock and hypotension (RUSH): learning outcomes and clinical performance in a proof-of-concept study
Source: BMC Med Educ. 2024 Apr 2;24:360. doi: 10.1186/s12909-024-05331-3 (PMC10988853; doi:10.1186/s12909-024-05331-3)
Supplement: Supplementary file 3 — Supplementary Material 3 [file 12909_2024_5331_MOESM3_ESM.docx]

**RUSHPRO Ultrasound View Rating Criteria**

**Anatomical structures that should be displayed in the respective position:**

Pos. 1) both ventricles, left atrium, aortic and mitral valve.

Pos. 2) both ventricles, both atria, tricuspid and mitral valve.

Pos. 3) right atrium (partially), inferior vena cava.

Pos. 4) pulmonal recessus, diaphragm, liver, Morrison’s pouch, kidney.

Pos. 5) pulmonal recessus, diaphragm, splen, Kollor’s pouch, kidney.

Pos. 6) bladder.

Pos. 7) aorta (cross-cut).

Pos. 8 + 9) two ribs (bat-sign), intercostal muscle, pleura.
