# Supplementary material for: Teaching Medical Students Rapid Ultrasound for shock and hypotension (RUSH): learning outcomes and clinical performance in a proof-of-concept study
Source: BMC Med Educ. 2024 Apr 2;24:360. doi: 10.1186/s12909-024-05331-3 (PMC10988853; doi:10.1186/s12909-024-05331-3)
Supplement: Supplementary file 5 — Supplementary Material 5 [file 12909_2024_5331_MOESM5_ESM.docx]

**RUSHPRO OSCE Checklist**

Participant No.

**Practical skills on the simulator (Performance Score)**

**Acquired views (fully: 2 points, partially: 1 point, not: 0 points)**

*not possible = anatomically or otherwise*

*(e.g., dressings) not possible to scan*

*🡪 reduces maximum achievable points*

Pos. 1) 🞏 fully 🞏 partially 🞏 not acquired 🞏 not possible

Pos. 2) 🞏 fully 🞏 partially 🞏 not acquired 🞏 not possible

Pos. 3) 🞏 fully 🞏 partially 🞏 not acquired 🞏 not possible

Pos. 4) 🞏 fully 🞏 partially 🞏 not acquired 🞏 not possible

Pos. 5) 🞏 fully 🞏 partially 🞏 not acquired 🞏 not possible

Pos. 6) 🞏 fully 🞏 partially 🞏 not acquired 🞏 not possible

Pos. 7) 🞏 fully 🞏 partially 🞏 not acquired 🞏 not possible

Pos. 8) 🞏 fully 🞏 partially 🞏 not acquired 🞏 not possible

Pos. 9) 🞏 fully 🞏 partially 🞏 not acquired 🞏 not possible

| **Achievable points** | **Achieved points** | **Percentage** | **Performance time [min:sec]** |
| --- | --- | --- | --- |
|  |  |  |  |

Participant No.

**Diagnostic skills on the simulator**

| **Case/Pathology:** |  |
| --- | --- |

*Scoring according to the simulators case preset.*

| **Achievable points** | **Achieved points** | **Percentage** |
| --- | --- | --- |
|  |  |  |

**Written documentation**

*Items to score: all views described, understandable, pathology adequately described*

🞏 Good (2 points) 🞏 Moderate (with shortcomings, 1 point) 🞏 Insufficient (0 points)

| **Totally achievable points** | **Achieved points** | **Percentage** |
| --- | --- | --- |
|  |  |  |
